# Advanced Grafting Biomaterials and Technologies in Chronic Wound Care: Mechanisms, Clinical Outcomes, and Therapeutic Integration

**DOI:** 10.3390/jfb17050239

**Published:** 2026-05-09

**Authors:** Albert D. Luong, Moorthy Maruthapandi, John H. T. Luong

**Affiliations:** 1 Innovative Wound Care (IWC), Fresno, CA 93710, USA; dluong.md@gmail.com; 2 Department of Chemistry, Bar-Ilan University, Ramat-Gan 52900, Israel; moorthm@biu.ac.il; 3 Bar-Ilan Institute for Nanotechnology and Advanced Material, Bar-Ilan University, Ramat-Gan 52900, Israel; 4 School of Chemistry, University College Cork, T12 YN60 Cork, Ireland

**Keywords:** bioengineered skin substitutes, placental-derived grafts, acellular dermal matrices, chronic wounds, wound healing mechanisms, advanced wound-care therapies, antimicrobial

## Abstract

Chronic wounds remain a major clinical and economic burden due to persistent inflammation, impaired perfusion, microbial biofilms, and dysregulated immune responses that collectively stall epithelialization. Polymicrobial bacterial–fungal biofilms, including *Candida* species, further delay healing by sustaining inflammation and promoting treatment-resistant infection. Recent advances have accelerated the development of bioengineered skin substitutes, collagen matrices, and placental-derived grafts that modulate macrophage polarization, reactive oxygen species signaling, and extracellular matrix remodeling to restore tissue architecture and promote neovascularization. Their effectiveness, however, depends on integration within structured care pathways that emphasize debridement, moisture balance, and infection control. Artificial intelligence, three-dimensional bioprinting, flexible microelectronic sensors for real-time wound monitoring, and bioactive compounds derived from traditional Chinese medicine, are expanding the therapeutic landscape. Together, these innovations support a shift toward predictive, personalized, and regenerative wound-care strategies. This review aims to provide a mechanistic and clinically contextualized overview of advanced grafting biomaterials, highlighting current applications, limitations, and future directions in chronic wound care.

## 1. Introduction

As a major and growing challenge for modern healthcare systems, chronic wounds (CWs) affect millions of individuals worldwide. They impose a substantial economic burden because of their prolonged treatment, recurrent infections, and high rates of hospitalization [1]. In the United States, CWs affect more than 8 million Medicare beneficiaries annually, with care costs exceeding tens of billions of dollars [2]. Globally, aging populations, diabetes, obesity, and vascular diseases continue to drive incidence upward [1,3]. Despite their differences in etiology, CWs share a common biological outcome. They fail to progress beyond inflammation, remaining stuck in a state marked by biofilm buildup, high protease activity, impaired macrophage polarization, and poor perfusion. This dysfunctional microenvironment prevents effective tissue repair and increases susceptibility to infection.

CWs reduce mobility, diminish quality of life, and significantly increase the risk of sepsis and amputation [1,3]. Infected patients often require repeated admissions and prolonged stays, resulting in high per-episode costs [3]. In clinical practice, the most frequently encountered wounds are diabetic foot ulcers (DFUs), venous leg ulcers (VLUs), pressure injuries (PIs), non-healing surgical wounds (NHSWs), and burn-related injuries [1,3], as summarized in Table 1. DFUs are particularly devastating, with a substantial proportion progressing to lower-limb amputation and higher long-term mortality rates, compared with several common cancers [1].

Complex wounds require aggressive debridement, biofilm disruption, ECM replacement, angiogenic stimulation, and immune modulation. These challenges can only be addressed by biological grafts, collagen dermal matrices, living skin substitutes, and negative pressure wound therapy [1]. Consequently, the market for advanced wound therapies has expanded rapidly; however, a persistent gap remains between clinical practice, biomaterial innovation, grafting technologies, and the economic realities that dictate their adoption.

This review focuses on an integrative framework linking biological mechanisms, clinical evidence, emerging technologies, and global health considerations to outline a more predictive, equitable, and regenerative future for wound management. It also highlights the increasing recognition of fungal involvement in CWs and the expanding clinical role of advanced grafting technologies. The discussion is extended to the transformative potential of emerging 3D bioprinting and AI in reshaping graft design, risk assessment, and personalized wound care, particularly within resource-limited settings.

As this review adopts a narrative (non-systematic) approach, it may be subject to selection bias in the literature considered. The authors sought to incorporate recent, clinically pertinent, and mechanistically insightful studies. Nevertheless, inherent limitations arise from inconsistencies in reporting standards, study design, and outcome measures among published publications. Therefore, interpretations and conclusions should be considered within the framework of these constraints.

## 2. Clinical Wound Healing Biology

Wound healing is a dynamic and highly coordinated physiological process essential for restoring tissue integrity following injury. This biological sequence proceeds through four overlapping phases: hemostasis, inflammation, proliferation, and remodeling. Each step of this process is driven by distinct cellular and molecular events [4]. Effective wound resolution necessitates timely progression through each stage; any interruption in this sequence often precipitates delayed healing or the development of chronic wound conditions.

### 2.1. Phases of Normal Wound Healing

Physiological wound healing occurs through a series of integrated phases. Hemostasis initiates immediately upon injury as platelets aggregate and generate a fibrin clot, which arrests hemorrhage and establishes a provisional ECM scaffold for recruited cellular populations [4]. During the inflammatory phase, neutrophils and monocytes infiltrate the wound site to clear necrotic debris and pathogens. Subsequently, macrophages become the predominant immune population and orchestrate the transition toward tissue repair [5]. During the proliferative phase of wound healing, fibroblasts play a central role by synthesizing the nascent extracellular matrix (ECM), which provides structural support and guides tissue regeneration. Concurrently, keratinocytes migrate to the wound surface and facilitate re-epithelialization, restoring the protective barrier of the skin. This coordinated activity ensures proper coverage and integration of new tissue within the wound site. Endothelial cells then drive angiogenesis to restore tissue perfusion. During the final remodeling phase, type III collagen is progressively replaced by type I collagen, enhancing tensile strength and facilitating ongoing scar maturation, a lengthy process that may persist for months or even years [4].

### 2.2. Impairment of the Inflammatory Transition in Chronic Wounds

**Figure 1:**
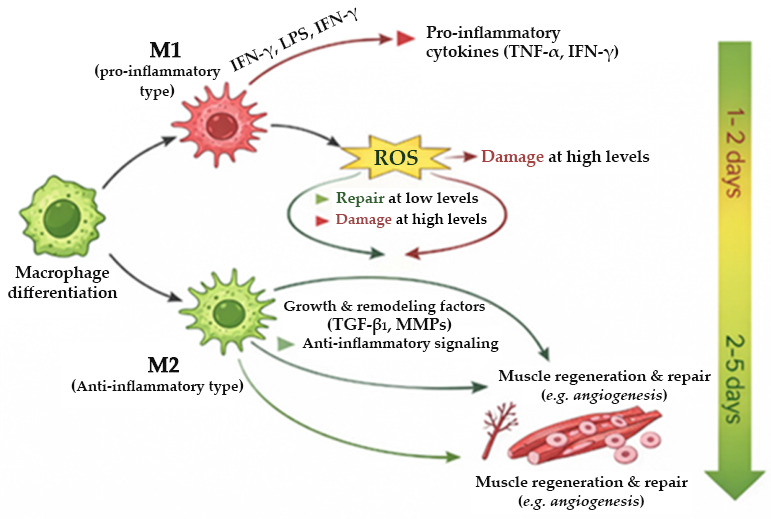
Temporal regulation of muscle injury and repair via macrophage polarization and ROS signaling. M1 macrophages generate robust ROS bursts that facilitate debris clearance while providing the redox cues necessary for satellite-cell activation. Following resolution of inflammation, M2 macrophages establish attenuated, pro-regenerative ROS levels that support satellite-cell proliferation and fusion, concurrently secreting mediators that coordinate collagen turnover and matrix reorganization. This temporal shift in macrophage phenotype orchestrates the ROS microenvironment and ECM dynamics required for efficient muscle regeneration. (Created with AI tools, original artwork by the authors).

Following injury, macrophages undergo a dynamic phenotypic shift from an early pro-inflammatory M1 state (days 1–2) to a pro-regenerative M2 state (days 2–5), representing a coordinated transition from inflammatory clearance to tissue restoration (Figure 1).

Excessive or prolonged M1 activity can aggravate tissue damage and induce apoptosis. As inflammation resolves, macrophages transition toward the M2 phenotype, characterized by the secretion of anti-inflammatory cytokines (e.g., TGF-β1), stem-cell recruitment signals (e.g., NAMPT), and matrix-associated factors (e.g., TAM-like cells and MMPs). M2 macrophages fulfill critical roles in promoting angiogenesis, regulating lipid metabolism, facilitating tissue remodeling, and supporting the regeneration of damaged structures. CWs typically fail to progress beyond the inflammatory phase due to a failure in the M1-to-M2 macrophage phenotypic shift [5,6]. Instead of transitioning to the proliferative phase, these wounds remain dominated by persistent inflammation, excessive protease activity, and impaired cellular signaling. Consequently, macrophages remain sequestered in an M1-dominant state, perpetuating tissue destruction and impeding organized matrix deposition [5].

### 2.3. Biofilm and Infection as Major Barriers to Resolution

Biofilm communities, identified in 60–80% of chronic wounds, sequester pathogens from antimicrobial agents, stimulate persistent inflammation, augment protease activity, and compromise macrophage functionality. The resulting microbial persistence reinforces the pro-inflammatory M1-dominant microenvironment, effectively arresting the wound in the inflammatory phase [5].

### 2.4. Perfusion and Oxygenation

Adequate perfusion is a prerequisite for healing, as oxygen drives essential processes, including fibroblast activity, collagen synthesis, angiogenesis, and microbial eradication. Chronic DFUs and VFUs frequently develop in ischemic and hypoxic tissues. In these environments, inadequate perfusion impairs metabolic and immune functions while sustaining pro-inflammatory macrophage activity [6]. Persistent M1-dominant inflammation, protease-rich exudate, biofilm recalcitrance, and ischemic hypoxia collectively establish a microenvironment incompatible with tissue regeneration. Even after biofilm disruption, the wound may still lack a competent ECM, angiogenic support, mechanical cues, and appropriate immunoregulation.

Advanced biomaterial-based grafts are engineered to rectify these deficits by restoring ECM architecture, sequestering excess proteases, and facilitating macrophage transition toward pro-healing M2 phenotypes. Their microscale and nanoscale topographies guide fibroblast migration and keratinocyte re-epithelialization, while tailored materials stabilize growth factors, enhance oxygen diffusion, and support neovascularization in hypoxic tissues, effectively reconstructing a functional regenerative niche.

## 3. Standard Treatment Modalities in Wound Care

CWs typically persist in a disrupted and prolonged inflammatory phase characterized by excessive protease activity, impaired perfusion, recalcitrant biofilms, and dysregulated moisture balance. Standard wound-care modalities are designed to overcome these underlying pathophysiological impediments by restoring a functional wound bed capable of granulation and re-epithelialization. Primary interventions encompass the removal of devitalized tissue, optimization of moisture levels, reduction in microbial burden, and mechanical or biochemical stimulation of tissue repair.

### 3.1. Debridement

Debridement facilitates the removal of devitalized tissue and biofilm, establishing a biologically active wound bed that supports granulation and epithelialization. Surgical debridement remains the most effective modality for rapidly eliminating necrotic tissue and reducing bacterial load [7]. Enzymatic approaches, typically utilizing collagenase, provide a selective alternative for patients who are poor surgical candidates [8]. Autolytic debridement employs occlusive or semi-occlusive dressings to activate endogenous enzymes and macrophages, thereby facilitating the gentle liquefaction of necrotic tissues [9]. Mechanical techniques, including wet-to-dry dressings and irrigation, effectively remove slough but may concurrently disrupt viable tissue. In CWs, biofilm-targeted debridement integrates serial sharp debridement with antimicrobial cleansing to disrupt biofilm architecture and minimize microbial density [10].

### 3.2. Moisture-Balance Dressings

Maintaining a moist wound environment facilitates accelerated epithelial migration, attenuates pain, and supports autolytic debridement [11]. Hydrogels rehydrate eschar and support autolysis, whereas hydrocolloids interact with exudate to form a gel that maintains humidity and offers thermal insulation. Alginate dressings are specifically designed to manage wounds with moderate-to-heavy exudate. Their unique composition allows them to effectively absorb excess fluid, helping to maintain optimal moisture levels within the wound bed. In addition to their absorptive properties, alginate dressings release calcium ions, which play a critical role in promoting hemostasis. By facilitating clot formation, these dressings help control bleeding and create a stable environment that supports the healing process. Foam dressings provide cushioning and excellent exudate management, whereas transparent films function as semi-permeable barriers appropriate for superficial wounds with minimal drainage [12].

### 3.3. Infection Control

Rigorous infection control is imperative to prevent biofilm reformation and facilitate tissue repair. Silver- and iodine-based dressings exhibit broad-spectrum antimicrobial activity [13]. Polyhexamethylene biguanide (PHMB) provides sustained bactericidal action with low cytotoxicity. Medical-grade Manuka honey promotes autolysis, exerts osmotic antimicrobial effects, and inhibits bacterial proliferation. Topical antibiotics should be utilized judiciously to mitigate the risk of resistance, whereas systemic antibiotics are reserved for cases of spreading infection, cellulitis, or osteomyelitis [10].

### 3.4. Negative Pressure Wound Therapy (NPWT)

NPWT and NPWT with instillation (NPWTi-d) utilize controlled sub-atmospheric pressure to augment perfusion, remove exudate, and stimulate the formation of granulation tissue. Their primary mechanisms include micro-deformation at the wound surface, reduction in interstitial edema, and mechanical stimulation of fibroblasts and endothelial cells [9]. NPWT is particularly efficacious in the management of DFUs, post-surgical wounds, and pressure injuries [14].

NPWT is widely used alongside grafting procedures to enhance graft adherence, stabilize the wound environment, and reduce fluid accumulation beneath the graft interface. NPWT enhances microvascular perfusion, reduces interstitial edema, and limits shear forces, thereby aiding graft integration and preventing early loss [15]. Early clinical experiences also demonstrated that subatmospheric pressure can limit burn wound progression and improve the quality of the wound bed prior to grafting [16]. More recent applications extend to complex infections and reconstructive scenarios, where NPWT combined with instillation has been shown to improve wound conditioning and facilitate subsequent graft or flap integration [17]. Overall, NPWT-assisted grafting generally yields better outcomes than grafts alone, although success remains dependent on wound characteristics, vascular status, and infection burden.

### 3.5. Adjunctive Therapies

Diverse adjunctive therapies are required to support healing when standard care protocols prove insufficient. Hyperbaric oxygen therapy enhances tissue oxygenation and leukocyte activity, while electrical stimulation facilitates angiogenesis and fibroblast proliferation. Ultrasound therapy promotes cell migration and collagen deposition, and laser therapy or photo-biomodulation attenuates inflammation while stimulating mitochondrial activity [18].

Although debridement, moisture regulation, infection control, and mechanical support can optimize the wound bed, persistent deficiencies in ECM integrity, angiogenesis, or immune regulation may still impede closure. In such instances, biological grafting represents a critical escalation in wound management by providing the requisite cellular, structural, and signaling components that the compromised wound environment can no longer produce endogenously.

## 4. Grafting: A High-Value Clinical Intervention

Chronic and complex wounds often require biological replacement strategies to re-establish tissue architecture and a functional healing environment. Grafting provides an advanced therapeutic option, capable of delivering cellular, ECM, and biochemical components that the wound bed cannot supply. Such interventions facilitate expedited wound closure, mitigate the risk of infection, enhance mechanical stability, and promote sustained functional recovery.

### 4.1. The Role of Grafting

DFUs, post-surgical dehiscence, burns, and pressure injuries [19], often fail to progress through normal healing phases due to infection, ischemia, or cellular senescence. Grafting is one of the most effective interventions for managing such complex, non-healing wounds. It provides biological coverage that promotes angiogenesis, granulation, and re-epithelialization [20,21]. Deep tissue loss and exposure of structures, such as tendon, bone, or joint capsule, frequently require grafting to restore protective barriers and prevent infection [22]. In burn care, grafting remains a cornerstone for rapid wound closure, reducing evaporative fluid loss, infection risk, and long-term scarring [23]. Chronic DFUs and VLUs similarly benefit from bioengineered or amniotic grafts that deliver ECM components and growth factors capable of reactivating stalled healing pathways [24].

Successful graft integration depends not only on material selection but also on meticulous post-application wound care. Important clinical steps are immobilizing the graft site, maintenance of moisture balance, preventing infection, and monitoring for early signs of issues like necrosis, hematoma, or detachment. Infection control remains particularly critical, as microbial burden and exudate accumulation can rapidly undermine graft viability [25]. Off-loading strategies for plantar wounds, compression therapy for venous ulcers, and strict glycemic control in diabetic patients are essential adjuncts that significantly influence graft survival and long-term outcomes [9,26]. The principles of postoperative management are critical factors influencing graft outcomes, yet they frequently receive insufficient attention in biomaterial-related discussions, despite their significant role in shaping wound healing progress.

### 4.2. Types of Grafts

Diverse graft materials are used to restore structure and biological function in chronic wounds, each differing in origin, composition, and regenerative potential. These materials span patient-derived autografts, human donor allografts, xenogeneic matrices from non-human sources, and bioengineered constructs designed to deliver structural support and targeted biological signals.

#### 4.2.1. Autografts

Autografts, harvested from the patient’s own tissue, remain the gold standard due to complete histocompatibility and minimal risk of immunologic rejection. Split-thickness skin grafts (STSGs), which include the epidermis and part of the dermis, are widely used for large surface wounds because donor sites re-epithelialize rapidly. Full-thickness skin grafts (FTSGs) provide greater durability and reduced contraction but require primary closure at the donor site [27]. Autografts are limited by donor-site morbidity and insufficient availability in extensive burns [28].

#### 4.2.2. Allografts

They are cadaveric skin or decellularized dermis that provides temporary biological coverage and serving as a scaffold for wound-bed preparation. They are valuable for massive burns, where they support vascularization and suppress bacterial proliferation [29]. Advanced decellularization techniques preserve ECM architecture while minimizing immunogenicity, improving graft integration and safety [18].

#### 4.2.3. Bioengineered Skin Substitutes

They represent a major advance in modern wound management, occupying the space between temporary dressings and fully autologous grafting. These constructs function as biologically active matrices designed to replicate key structural and signaling functions of native skin [21]. Tissue-engineering strategies have generated both cellular constructs with paracrine activity and acellular scaffolds to restore dermal architecture and guide host regeneration.

- Apligraf^®^ is a bilayer living skin equivalent composed of neonatal fibroblasts in a type I collagen matrix overlaid with human keratinocytes. It provides coordinated dermal support and epidermal signaling that accelerate re-epithelialization in DFUs and VLUs.
- Dermagraft^®^ contains human fibroblasts cultured on a bioabsorbable scaffold, delivering sustained ECM production and fibroblast-derived cues that promote granulation and angiogenesis in chronic DFUs.
- Integra^®^ is a bilayer dermal regeneration template with a collagen–GAG matrix beneath a temporary silicone layer, supporting vascular ingrowth and neodermis formation. It is particularly valuable for deep burns and full-thickness defects.
- PriMatrix^®^, derived from the fetal bovine dermis, preserves native ECM ultrastructure to support cell infiltration, rebalance inflammatory signaling, and promote neovascularization in chronic and post-surgical wounds [21].

Collectively, these constructs enhance the closure of diabetic, venous, traumatic, and surgical wounds and often outperform standard dressings when integrated into comprehensive, multimodal care [20].

Table 2 illustrates differences in composition, processing, mechanisms, and evidence strength rather than implying a therapeutic hierarchy, a distinction that is often overlooked in clinical discussions and even in some published reviews [30,31,32,33,34]. Apligraf^®^ and Dermagraft^®^ are supported by multiple Level I randomized controlled trials showing superior closure rates over standard care, whereas Integra^®^ and PriMatrix^®^ have demonstrated value in deeper or structurally complex wounds through strong prospective and cohort data. In both preclinical and clinical studies, including the retrospective analysis by Paredes et al. [34], PriMatrix^®^ achieved ~70% VLU closure within 12 weeks with enhanced granulation and epithelialization.

Overall, no universal ranking among grafts is supported; optimal selection depends on wound depth, tissue loss, inflammation, vascular status, and care setting. When appropriately matched to wound biology, these matrices reduce repeated debridement, accelerate healing, and improve long-term outcomes. The evidence levels in Table 2 indicate the relative strength of clinical support, with Level I representing the highest-quality data. Furthermore, Table 3 provides an expanded overview of the structural organization, cellularity, and biomaterial design features of major commercial grafts, emphasizing how differences in scaffold architecture and cell content shape their regenerative mechanisms and clinical applications.

#### 4.2.4. Placental and Amniotic Grafts

Amniotic and chorionic membranes are naturally rich in growth factors, hyaluronic acid, and collagen, providing anti-inflammatory, anti-fibrotic, and pro-regenerative benefits [23]. Placental and amniotic grafts form a distinct class of biologically active matrices that harness the innate regenerative properties of fetal tissues to rebalance inflammatory signaling and accelerate healing. EpiFix^®^, Grafix^®^, AmnioExcel^®^, and PalinGen^®^ are available in dehydrated or cryopreserved forms and retain key placental components, including growth factors, hyaluronic acid, and ECM proteins. These grafts provide an active surface that regulates inflammation, supports cell migration, and promotes angiogenesis, whereas their anti-fibrotic and antimicrobial properties stabilize the wound bed. They are particularly effective for chronic DFUs, VFUs, and radiation-induced soft-tissue injuries.

Clinical studies consistently demonstrate that placental-derived grafts reduce healing time compared with standard care, often achieving faster granulation, earlier re-epithelialization, and fewer required applications [24]. The commercial products summarized in Table 4 only exhibit overlapping indications and biological functions; comparative features are presented to support informed selection rather than to establish clinical priority.

As summarized in Table 4, Grafix^®^ and EpiFix^®^ are supported by Level I RCTs, providing the strongest evidence for accelerated wound closure and infection reduction [24,39]. AmnioExcel^®^, supported by prospective clinical trial data [40], has demonstrated significant improvement in healing rates compared with standard care alone. PalinGen^®^, now backed by peer-reviewed clinical evidence [41], has shown effective granulation and closure in pressure ulcers using a dual-layer amniotic allograft. Among the four cryopreserved matrices, Grafix^®^ and PalinGen^®^ preserve viable cells and regenerative signaling, whereas dehydrated membranes like EpiFix^®^ and AmnioExcel^®^ offer longer shelf life and easier handling. All four matrices demonstrate measurable benefits in accelerating healing, reducing infection, and supporting tissue regeneration, underscoring their value as advanced biological options within standard wound-care protocols. Although these placental-derived grafts differ in processing, cellular viability, and matrix composition, current evidence does not justify prioritizing one preparation universally across all wound types. When integrated into a structured wound-care algorithm, they can shorten healing trajectories, reduce recurrence, and provide an effective option for wounds unresponsive to conventional therapy.

Grafting remains a high-value, evidence-based intervention bridging traditional surgery and regenerative medicine. From autologous grafts to bioengineered and placental substitutes, each modality offers distinct biological and structural advantages tailored to wound depth, chronicity, and patient comorbidities. Emerging regenerative platforms using stem cells, growth factors, and advanced biomaterials may further enhance graft integration and outcomes [28].

#### 4.2.5. Synthetic and Biodegradable Dermal Matrices

Biodegradable synthetic dermal matrices have become important alternatives to biological grafts, offering controlled structural properties, predictable degradation, and reduced variability compared with tissue-derived substitutes. Of note is the biodegradable temporizing matrix (BTM), a synthetic polyurethane bilayer scaffold designed to enable cell growth, blood vessel formation, and new skin development. BTM offers a stable, infection-resistant structure for complex wounds, such as burns, trauma, and non-graftable defects, where biological matrices may not be suitable [42,43]. Recent multicenter studies have demonstrated that synthetic dermal matrices integrate reliably with various complex wounds, providing consistent results and lower immunogenic risk [44]. Recent reviews highlight the increasing application of acellular and synthetic substitutes in staged reconstruction, attributing their adoption to customizable mechanical properties, consistent degradation profiles, and compatibility with adjunctive therapies [45]. Integration of synthetic matrices with bioactive systems like chitosan–curcumin may lead to hybrid wound therapies that offer structural support along with antimicrobial, antifungal, and immunomodulatory benefits.

## 5. Mechanisms of Action

**Figure 2:**
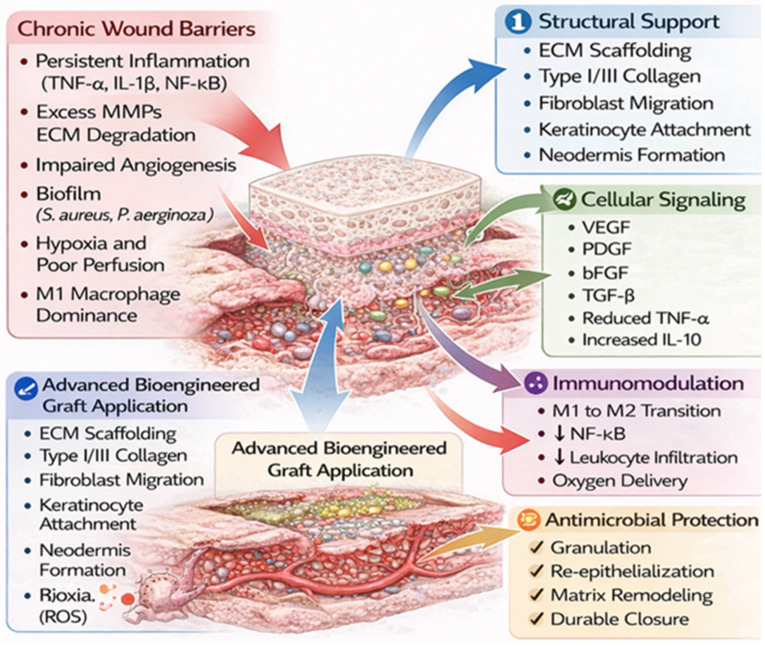
Grafts facilitate wound resolution through: (i) structural support via 3D ECM scaffolding and type I/III collagen architecture, promoting fibroblast migration and keratinocyte attachment; (ii) cellular signaling mediated by growth factors, including VEGF, PDGF, bFGF, and TGF-β, which drive proliferation, angiogenesis, and re-epithelialization; (iii) immunomodulation, characterized by the M1-to-M2 macrophage phenotypic transition and suppression of NF-κB-mediated inflammatory pathways; (iv) angiogenesis, enhancing endothelial sprouting and microvascular stabilization; and (v) antimicrobial and protease-modulating activity that limits biofilm persistence and ECM degradation. (Created with BioRender.com; original artwork by the authors).

Advanced grafts facilitate accelerated wound healing through synergistic structural, biochemical, and immunomodulatory mechanisms. These pathways directly address the biological impediments characteristic of chronic wounds, such as persistent inflammation, excessive protease activity, impaired angiogenesis, and ECM degradation (Figure 2).

### 5.1. Structural Support

Numerous grafts provide a 3D-ECM scaffold that replicates native dermal architecture, thereby supporting fibroblast migration, keratinocyte attachment, and organized collagen deposition as the wound transitions from the inflammatory to the proliferative phase [46]. This structural framework stabilizes the wound bed, mitigates mechanical shear, and serves as a template for neodermis formation. Collagen-based grafts supply a type I/III fibrillar scaffold that resists enzymatic degradation, directs fibroblast organization, and enhances tensile strength during the remodeling phase [47]. Furthermore, the preserved ultrastructure promotes angiogenesis by providing a substratum for endothelial cell migration.

### 5.2. Cellular Signaling

Bioengineered and placental grafts modulate reparative processes through various signaling pathways by sequestering or stabilizing essential growth factors, including VEGF, PDGF, TGF-β, and bFGF. Consequently, these grafts are designed to stimulate fibroblast proliferation, endothelial sprouting, and keratinocyte migration, thereby accelerating granulation tissue formation and re-epithelialization [48]. These matrices also modulate wound-bed cytokine profiles by attenuating pro-inflammatory mediators (e.g., TNF-α, IL-1β) and augmenting anti-inflammatory signals (e.g., IL-10, TGF-β1, etc.), restoring a regenerative microenvironment [49]. A pivotal mechanism involves macrophage polarization, as CWs often remain in a pro-inflammatory M1-dominant state. Placental, fetal-derived, and decellularized matrices promote transition toward the pro-reparative M2 phenotype, supporting angiogenesis and matrix deposition [5].

### 5.3. Anti-Inflammatory Effects

Placental and amniotic grafts demonstrate potent anti-inflammatory properties mediated by cytokines, hyaluronic acid derivatives, and immunomodulatory proteins that suppress chronic inflammation [50]. These biomaterials reduce leukocytic infiltration, inhibit NF-κB activation, and disrupt the self-sustaining inflammatory loops typical of recalcitrant wounds. Additionally, collagen grafts function as sacrificial substrates for matrix metalloproteinases (MMPs), shielding endogenous ECM and facilitating fibroblast-mediated dermal repair [51].

### 5.4. Angiogenesis

Many grafts inherently contain or induce the secretion of angiogenic growth factors, such as VEGF, PDGF, and bFGF, which stimulate endothelial cell proliferation, lumen formation, and stabilization of nascent microscale vessels [52]. By mitigating edema, modulating inflammation, and supporting neovascularization, these materials enhance local perfusion, a critical therapeutic benefit in ischemic diabetic and venous ulcers, where compromised blood flow represents a primary barrier to resolution [53].

### 5.5. Antimicrobial Effects

Certain biological grafts exhibit inherent antimicrobial activity through natural host-defense peptides, such as defensins and cathelicidins, which disrupt bacterial membranes and impede biofilm formation [54]. Amniotic and chorionic membranes provide innate immune factors that constrain bacterial proliferation with minimal immunogenicity [55]. Their integrated structural, biochemical, immunomodulatory, angiogenic, and antimicrobial actions address the fundamental deficits of chronic wounds and are increasingly evolving toward more sophisticated regenerative designs.

### 5.6. Microbiology and Antimicrobial Stewardship in Chronic Wounds

*Staphylococcus aureus* (including MRSA) and *Pseudomonas aeruginosa* remain the most prevalent pathogens isolated from chronic wounds, with frequently biofilm-mediated persistence and healing delays. Empiric therapy typically comprises β-lactam/β-lactamase inhibitors, fluoroquinolones, glycopeptides, or carbapenems; however, antimicrobial resistance is widespread. MRSA accounts for 20–40% of *S. aureus* isolates, whereas multidrug-resistant *P. aeruginosa* is identified in up to 30% of cases [56,57,58,59,60]. DFUs and VFUs are characteristically polymicrobial, whereas burns and pressure injuries more frequently harbor highly resistant MRSA and *Acinetobacter baumannii*. Anaerobic bacteria and fungi, though less frequently identified, contribute significantly to the recalcitrance of ischemic wounds. Biofilms, present in ~ 80% of chronic wounds, represent a formidable barrier to resolution, necessitating aggressive surgical debridement, rigorous antimicrobial stewardship, and the application of advanced dressings [58,59,60].

Table 5 delineates key pathogens, standard therapeutic regimens, resistance profiles, and clinical success rates across major wound categories. With optimized debridement and wound-bed preparation, targeted antimicrobial therapy achieves success rates of 70–80% in most instances. However, outcomes are highly contingent upon pathogen sensitivity, effective biofilm disruption, and the quality of local wound management. Empiric therapy should be refined expeditiously using culture-directed antibiotics to mitigate treatment failure and limit the emergence of resistance. Polymicrobial, biofilm-rich wounds often require a combination of systemic and topical agents, supported by serial debridement and adjunctive modalities such as NPWT to enhance antibiotic penetration and therapeutic efficacy.

## 6. Economic Considerations in Advanced Wound Care

DFUs, VLUs, pressure injuries, and burns incur a significant economic burden due to delayed healing and infection [57,58,59,60]. In the United States, 6.5 million patients suffer from $28–$31 billion in annual costs; DFU admissions average $20,000–$30,000, and amputations exceed $60,000–$75,000 [61,62]. Delayed closure, recurrent infection, diabetes, venous insufficiency, and biofilm-driven inflammation are the principal cost drivers, with chronic wounds consuming 2–4% of national healthcare budgets. Each additional month without healing incurs $1600–$2400 in outpatient costs [58,60,61].

Although advanced grafts increase upfront spending, bioengineered Apligraf^®^ and Dermagraft^®^ shorten healing by 30–50%, reducing total episode-of-care costs by 20–25%. NPWT further decreases dressing frequency, infection, and readmissions, saving $3200–$5000 per patient [63,64]. Reimbursement policies strongly influence utilization; in the U.S., payers require documentation of debridement, infection control, and failure of standard care, while single-use NPWT systems abroad save £1200–£1500 ($1500–$1900) per surgical wound [65].

Economic analyses consistently show early use of bioengineered and placental grafts to be cost-effective, with placental matrices averaging $6800 per healed wound versus $11,000 for moist wound therapy [63]. Indirect societal costs add $10–$15 billion annually through disability, lost productivity, and long-term care. Timely advanced interventions shorten hospital stay by 3–5 days, reduce readmissions by up to 25%, and prevent deep infection or amputation, yielding substantial downstream savings [62].

## 7. Clinical Outcomes and Real-World Effectiveness

Advanced grafts and bioengineered matrices successfully accelerate healing, with meta-analyses showing 40–60% higher rates of complete closure and healing occurring 4–6 weeks faster than standard care [30]. Apligraf^®^ and Dermagraft^®^ improve 12-week DFU healing by 30–56%, Integra^®^ achieves up to 90% graft take with reduced contracture, and dehydrated amnion/chorion allografts reach 60–85% closure in DFUs and VLUs within 8–12 weeks [66,67,68,69]. Placental matrices further rebalance inflammatory signaling, enhance antimicrobial peptide release, and promote fibroblast migration and angiogenesis, lowering infection-related complications [61]. Functional outcomes also improve, with shorter rehabilitation, better scar quality, and 25–40% gains in quality-of-life metrics [64]. Long-term durability is superior, with recurrence below 20% at 1 year for Apligraf and amniotic allografts versus 40–50% under conventional care [62].

Integrated protocols, including debridement, moisture balance, off-loading or compression, infection control, and NPWT, add another 20–30% improvement in closure and graft adherence [70,71]. Emerging evidence highlights fungi (*Candida*, dermatophytes, and molds) as major contributors to DFU chronicity, delaying healing and synergizing with bacterial biofilms [72,73,74,75]. Their role in diabetic foot osteomyelitis and frequent under-detection by routine culture support the need for routine fungal diagnostics and targeted therapy [76]. Mixed bacterial–fungal communities show higher antimicrobial tolerance and worse outcomes [77,78,79,80], and molecular profiling reveals fungal DNA in far more DFUs than culture detects, indicating a distinct clinical phenotype requiring integrated antifungal evaluation to prevent severe complications [78].

### Limitations of Clinical Evidence and Translational Challenges

Despite encouraging clinical outcomes, the evidence base for advanced grafting biomaterials remains heterogeneous. Clinical studies differ substantially in design, patient populations, wound etiology, endpoints, and follow-up duration, making direct comparison across therapies challenging. Many investigations rely on small cohorts or non-randomized designs, limiting the strength and generalizability of clinical conclusions. Translational challenges also persist for emerging biomaterials. Key barriers include manufacturing scalability, batch-to-batch reproducibility, sterilization constraints, and uncertainties regarding long-term safety. Regulatory pathways for advanced constructs—particularly those incorporating living cells, growth factors, or AI-assisted design—remain complex and may delay clinical adoption.

Economic considerations further influence real-world implementation. Although advanced grafts may reduce long-term healthcare costs by accelerating healing and preventing complications, their high upfront costs can limit accessibility, especially in resource-constrained settings. Reimbursement policies, infrastructure limitations, and clinician familiarity also shape adoption patterns. The diversity of chronic wounds and patient factors, such as vascular issues, metabolic disease, infection, and biomechanical stress, restricts the generalizability of clinical results. Advances depend on robust trials, standardized outcomes, and frameworks that assess biological efficacy alongside clinical practicality and cost.

## 8. Perspectives and Future Possibilities

### 8.1. 3D Printing and Artificial Intelligence in Next-Generation Skin Grafts

Next-generation grafts are moving toward patient-specific constructs tailored to the anatomical and inflammatory profiles of individual wounds. Advances in digital fabrication and computational modeling, particularly through the integration of 3D printing and AI, enable precise control over scaffold geometry, porosity, and material distribution, producing constructs that conform more accurately to wound topology [81,82,83,84,85,86,87,88,89,90,91,92,93,94]. Digital light processing (DLP) printing can generate tunable silk-fibroin- and other bioink-based skin models with dermal- and epidermal-like architectures, while reproducible workflows allow spatial placement of cells and bioactive elements [82,83,84,88,93,94].

AI enhances this process by classifying inflammatory phenotypes in dermatologic imaging, supporting more precise wound phenotyping and graft selection [87]. Machine learning models identify relationships among scaffold composition, mechanics, and cellular responses, enabling the optimization of bioink formulations and degradation kinetics [81,89,90,91,92]. Coupled with 3D wound bioimaging, AI-assisted modeling can generate custom-fit scaffolds that improve anatomical conformity, reduce suturing, and enhance tissue integration, including region-specific tuning for ischemic or hyperinflammatory ulcers [85,90,92].

Generative AI improves DFU image classification and segmentation; automated documentation reduces clinical workload; and decision-support algorithms assist in personalized treatment planning. Predictive models estimate healing trajectories and identify patients at risk for deterioration, while smartphone-based AI platforms support remote monitoring and triage, expanding access to care in resource-limited settings [95,96]. 3D bioprinting further enables multilayered constructs with strategic placement of cells and signaling molecules, while AI-driven optimization of stiffness, porosity, and degradation supports improved fibroblast, endothelial, and immune responses [81,82,83,84,85,86,87,88,89,90,91,92,93,94]. Iterative learning from clinical outcomes may ultimately yield adaptive, patient-specific regenerative designs.

Despite these advances, translation remains limited by manufacturing reproducibility, sterility, regulatory hurdles, vascular integration, and the need for high-quality datasets. Clinically viable adoption will require integrated workflows linking imaging, modeling, printing, and surgical application. In developing nations, low-cost 3D printers and AI-enabled smartphone diagnostics may have an even greater impact by decentralizing fabrication and improving access to wound assessment and treatment guidance [97,98,99,100,101]. Together, these technologies provide a pathway toward more equitable, data-informed, and personalized wound-care delivery.

Particularly, DFUs, precipitated by neuropathy, elevated plantar pressures, and shear forces, remain the primary etiology of non-traumatic lower-limb amputations, affecting up to 24% of neuropathic patients [102,103]. Although timely offloading and revascularization can facilitate healing [104,105], recurrence rates approach 60% within 3 years [106], imposing a significant clinical and economic burden. *Candida* and mixed bacterial–fungal biofilms play a major role in chronic wounds by causing delayed healing, deeper tissue involvement, and higher risks of necrosis and amputation [72,73]. Fungal pathogens hinder epithelialization, worsen inflammation, and create biofilms that increase virulence and antimicrobial resistance [74,75]. Recent research also links them to diabetic foot osteomyelitis [76]. This distinct fungal-associated phenotype necessitates the integration of routine fungal diagnostics and antifungal management strategies.

### 8.2. Flexible Electronics

Flexible and wearable electronic systems are emerging as powerful tools in modern wound care by enabling real-time monitoring of key physiological parameters, such as pH, oxygenation, temperature, and exudate composition. Recent advances in biocompatible printed electronics have further expanded these capabilities, allowing sensor arrays, bioresponsive patches, and textile-based drug-delivery dressings to be integrated directly into wound dressings for continuous and non-invasive assessment [107,108,109]. These platforms can detect early signs of infection or inflammation, supporting timely clinical intervention—an essential advantage in chronic wounds. The convergence of flexible electronics with bioactive materials also opens the possibility of “sense-and-respond” dressings capable of autonomously monitoring wound status and release therapeutics as needed. Such hybrid systems represent a significant step toward more accurate, responsive, and sustainable wound-care technologies [110].

### 8.3. Traditional Chinese Medicine (TCM)

Traditional Chinese medicine offers bioactive phytochemicals like curcumin, berberine, baicalin, asiaticoside, and paeoniflorin, which have antimicrobial, antioxidant, and immunomodulatory effects useful for chronic wound healing. TCM-derived compounds can modulate immune responses, regulate the wound microbiome, and promote angiogenesis and re-epithelialization when incorporated into advanced biomaterials [111,112]. Hydrogels and self-assembled bioactive materials derived from herbal extracts have demonstrated enhanced hemostasis, improved fibroblast activity, and accelerated closure of diabetic wounds [113,114]. These mechanistic pathways closely mirror numerous intracellular and redox-modulating effects associated with curcumin, indicating considerable potential for synergistic integration.

Future research should explore whether chitosan–curcumin systems can be combined with other TCM phytochemicals to achieve complementary antimicrobial, antifungal, and immunoregulatory effects. East–West therapeutic integration could support multimodal wound-healing platforms, but necessitates thorough assessment of solubility, stability, cytotoxicity, and pharmacodynamics to guarantee safety and consistency [115,116]. Nevertheless, a recent peer-reviewed case report demonstrated the potential value of integrating TCM with Western wound-care practices in refractory DFUs [117]. In a 57-year-old patient with Wagner grade IV DFU, the patient was unresponsive to standard antibiotics and debridement. Chinese herbal fumigation and acupuncture, combined with Western antimicrobial therapy, led to quick healing and full closure of the wound within 3 months, with no recurrence at 6 months. This case shows that TCM can improve circulation and lower inflammation, working alongside Western treatments for limb preservation in complex DFUs, particularly in resource-limited settings.

TCM has continued to gain relevance in chronic wound management through its diverse repertoire of bioactive phytochemicals and multimodal therapeutic strategies. Many TCM-derived compounds exhibit antimicrobial, anti-inflammatory, angiogenic, and immunomodulatory properties that complement contemporary wound-care approaches. Herbal formulations, topical extracts, and adjunctive therapies such as fumigation and acupuncture have demonstrated potential to enhance perfusion, modulate macrophage polarization, and accelerate granulation in refractory wounds, including diabetic foot ulcers. Increasing integration of TCM principles with modern wound models and biomaterial platforms highlights its value as a complementary therapeutic domain within regenerative wound care [118].

## 9. Concluding Remarks

Advanced grafts and bioengineered matrices address the physiological and structural deficits that impede chronic wound closure. Their ability to accelerate healing, reduce complications, and improve functional outcomes positions them as essential components of modern wound-care pathways, particularly as value-based models emphasize early, targeted intervention. Placental grafts, bioactive matrices, and engineered dermal templates offer more predictable results in complex wounds, and future progress will depend on aligning biomaterials science with clinical practice and economic feasibility. Within this evolving landscape, 3D bioprinting and AI are reshaping graft development. Bioprinting enables anatomically precise, multilayered constructs with tunable properties, while AI-driven imaging and predictive modeling support personalized graft selection and scaffold optimization. Together, these technologies shift graft fabrication from standardized manufacturing toward precision-engineered, data-guided regenerative solutions. Flexible microelectronics and bioactive compounds derived from TCM further broaden this trajectory. Wearable microelectronic sensors enable continuous monitoring of perfusion, inflammation, and exudate dynamics, supporting earlier intervention and more responsive graft management. Likewise, TCM-derived phytochemicals with antimicrobial, angiogenic, and immunomodulatory properties offer potential as synergistic biologic inputs within engineered matrices. Together, these innovations reinforce a multimodal, integrated approach to chronic wound care that complements next-generation graft technologies.

## Figures and Tables

**Table 1:** An overview of major chronic wound types and their clinical impact.

Wound Type	Primary Causes	Key Biological Barriers	Clinical Consequences
DFUs	Neuropathy, ischemia, pressure	Biofilm, impaired immunity, high protease activity	Infection, osteomyelitis, amputation
VLUs	Venous hypertension, edema	Chronic inflammation, fibrin cuffs, poor oxygenation	Recurrent wounds, long healing times
PIs	Prolonged pressure, immobility	Tissue ischemia, necrosis	Infection, hospitalization
NHSWs	Poor perfusion, infection, tension	Biofilm, dehiscence, ECM degradation	Delayed closure, reoperation
Burn Wounds	Thermal injury	Loss of dermis, infection risk, fluid imbalance	Grafting requirement, scarring

**Table 2:** Comparative Overview of Commercial Bioengineered and Collagen-Based Grafts (Table created by the authors based on representative clinical evidence findings reported in studies).

Product	Primary Mechanism of Action	Clinical Indications	Representative Clinical Evidence	Evidence Level
Apligraf^®^ (Organogenesis Inc., Canton, MA, USA)	Delivers living cells that secrete cytokines and growth factors; stimulates keratinocyte migration and re-epithelialization	DFUs and VLUs; post-surgical wounds	RCTs show 56–63% complete closure by 12 weeks vs. ~25% with SOC [30]	Level I (RCT)
Dermagraft^®^ (Organogenesis Inc., San Diego, CA, USA)	Sustained secretion of ECM proteins and growth factors; promotes granulation and angiogenesis	Chronic DFUs; refractory VLUs	Multicenter RCTs show ≈60% DFU closure by 12 weeks vs. ~25% with SOC [31]	Level I (RCT)
Integra (Integra LifeSciences, Princeton, NJ, USA)	Serves as a neodermal template, supports vascular ingrowth, minimizes contraction; overlaid with autograft after vascularization	Burns, trauma, full-thickness defects, chronic surgical wounds	Case series and clinical cohort studies show rapid neodermis formation and durable closure in deep burns and trauma [32,33]	Level II–III (prospective and case)
PriMatrix^®^ (Integra LifeSciences)	Provides a bioactive scaffold for cell infiltration, modulates inflammation, and promotes neovascularization	Chronic wounds, VLUs, post-surgical defects	Clinical series and retrospective studies report ~70% closure in VLUs by 12 weeks, with granulation and re-epithelialization [34]	Level II–III (prospective and comparative)

**Table 3:** Structural and Biomaterial Characteristics of Commercially Available Grafts (Table created by the authors based on specified structural features of commercial grafts).

Product/Ref.	Composition/Type	Cellularity	Key Structural Features	Functional Design Purpose
Apligraf^®^ [35]	Bilayer living skin equivalent (epidermal + dermal analog)	Living (keratinocytes + neonatal fibroblasts	Thin keratinocyte epidermal layer over a fibroblast-populated bovine collagen matrix	Mimics native skin architecture; delivers cytokines and growth factors; supports re-epithelialization
Dermagraft^®^ [36]	Fibroblast-seeded bioabsorbable scaffold	Living (human fibroblasts	Single-layer bioabsorbable polymer mesh with dispersed fibroblasts	Provides sustained ECM production; enhances granulation and dermal regeneration
Integra^®^ Dermal Regeneration Template [37]	Bilayer matrix (silicone epidermal analog + collagen–GAG dermal layer)	Acellular	Thin silicone top layer over porous collagen–GAG scaffold forming a neodermal template	Creates a stable dermal substitute; supports vascular ingrowth; reduces contracture
PriMatrix^®^ [38]	Fetal bovine dermal scaffold	Acellular	Preserved ECM architecture with intact collagen bundles	Provides a bioactive scaffold for cell infiltration and modulation of inflammation

**Table 4:** Comparative Overview of Commercial Placental/Amniotic Grafts Used in Chronic Wound Healing (Table created by the authors based on representative clinical evidence findings reported in studies).

Product	Formulation/Preservation	Key Biologic Features	Clinical Results	Best-Supported Indications
EpiFix^®^ (MiMedx Group, Inc.)	Dehydrated human amnion/chorion membrane (dHACM)	Retains growth factors (VEGF, PDGF, TGF-β, and EGF), structural ECM proteins, and anti-inflammatory cytokine	In an RCT involving 97 subjects, 92% of DFUs achieved complete closure by 6 weeks with EpiFix^®^ + SOC vs. 8% with SOC alone [24].	DFUs, VLUs, post-surgical and traumatic wounds
Grafix^®^ (Osiris Therapeutics/Smith + Nephew	Cryopreserved viable human placental membrane (vHPM)	Retains growth factors (VEGF, PDGF, TGF-β, and EGF), structural ECM proteins, and anti-inflammatory cytokines	In a multicenter RCT (*n* = 97), 62% of DFUs healed within 12 weeks vs. 21% with SOC (*p* = 0.0001) [39]. Mean time to closure: 42 days vs. 69 days.	Complex DFUs, post-surgical dehiscence, tendon/bone exposure
AmnioExcel^®^ (Integra LifeSciences)	Dehydrated human amniotic membrane (single-layer, acellular)	Structural scaffold preserving collagen, fibronectin, and growth factors; supports epithelial migration and angiogenesis	In a 2024 study, 45.5% of subjects treated with AmnioExcel^®^ + SOC achieved complete closure by 6 weeks, vs. 0% with SOC alone [40].	Chronic wounds (DFUs, VLUs, post-surgical dehiscence)
PalinGen^®^ (Amnio Technology, LLC)	Cryopreserved or dehydrated amniotic/chorionic membrane (available as membrane, particulate, or flow formulations)	Preserves ECM, hyaluronic acid, and native growth factors (VEGF, PDGF, and TGF-β); modulates inflammation and promotes angiogenesis	In a clinical case report of a posterior-thigh pressure ulcer, dual-layer amniotic membrane allograft promoted granulation and complete closure within 12 weeks, demonstrating durable wound coverage [41].	Pressure ulcers, DFUs, VLUs, and post-surgical or ischemic wounds

**Table 5:** Common Pathogens, Antibiotic Therapy, and Resistance Outcomes by Wound Type (Table created by the authors based on representative clinical results reported in studies).

Wound Type	Dominant Pathogens	Common Antibiotics Used	Resistance Patterns (%)	Clinical Success (%)/Refs.
DFUs	*S. aureus*, *P. aeruginosa*, *E. faecalis*, *E. coli*	Amoxicillin–Clavulanate, Piperacillin/Tazobactam, Vancomycin, Ciprofloxacin	MRSA 25%, ESBL 15%, *Pseudomonas* 20%	70% [56]
VLUs	*S. aureus*, *P. aeruginosa*, *E. faecalis*	Coamoxiclav, Doxycycline, Ciprofloxacin	MRSA 20%, *Pseudomonas* 40%	75% [57]
NHSW *	*S. aureus (MRSA)*, *E. faecalis*, *P. aeruginosa*	Vancomycin, Linezolid, Piperacillin/Tazobactam, Carbapenems	MRSA 40%, MDR *Pseudomonas* 25%	70% [58]
Burn Wounds	*S. aureus (MRSA)*, *P. aeruginosa*, *A. baumannii*	Ceftazidime, Meropenem, Colistin, Silver sulfadiazine	MRSA 40%, Carbapenem- resistant *Acinetobacter* 60%	80% [59]
PIs **	*S. aureus*, *P. aeruginosa*, *Enterococcus* spp., *Proteus* spp.	Amoxicillin/Lavulanate, Doxycycline, Vancomycin	MRSA 35%, MDR *Pseudomonas* 30%	70% [60]

* NHSW = Non-Healing Surgical Wounds; ** PIs = Pressure Injuries.

## Data Availability

No new data were created or analyzed in this study. Data sharing is not applicable to this article.

## Abbreviations

The following abbreviations are used in this manuscript:

AMP	Antimicrobial Peptide
bFGF	Basic Fibroblast Growth Factor
DFUs	Diabetic Foot Ulcers
ECM	Extracellular Matrix
ESBL	Extended-spectrum β-lactamase
FTSG	Full-Thickness Skin Graft
GAG	Glycosaminoglycan
IFN-γ	Interferon Gamma
IL	Interleukin
LPS	Lipopolysaccharides
M1	Classically Activated Macrophage Phenotype
M2	Alternatively Activated (Pro-Regenerative) Macrophage Phenotype
MDR	Multidrug-Resistant
MMP	Matrix Metalloproteinase
MRSA	Methicillin-resistant Staphylococcus aureus
NAMPT	Nicotinamide Phosphoribosyltransferase
NF-κB	Nuclear Factor Kappa B
NPWT	Negative Pressure Wound Therapy
NPWTi-d	Negative Pressure Wound Therapy with Instillation
PHMB	Polyhexamethylene Biguanide
PI	Pressure Injury
PDGF	Platelet-Derived Growth Factor
ROS	Reactive Oxygen Species
RCT	Randomized Controlled Trial
SOC	Standard of Care
STSG	Split-Thickness Skin Graft
TAM	Tumor-Associated Macrophage
TGF-β	Transforming Growth Factor Beta
TIMP	Tissue Inhibitors of Metalloproteinase
TNF-α	Tumor Necrosis Factor Alpha
VEGF	Vascular Endothelial Growth Factor
VLUs	Venous Leg Ulcers
